# Temporal Trends and Determinants of Gestational Diabetes Mellitus and Perinatal Outcomes in Tuscany, Italy (2010–2021)

**DOI:** 10.3390/jcm15145611

**Published:** 2026-07-17

**Authors:** Giuseppe Seghieri, Elisa Gualdani, Paolo Francesconi, Flavia Franconi, Graziano Di Cianni

**Affiliations:** 1 Epidemiology Unit, Agenzia Regionale Sanità, 50141 Florence, Italypaolo.francesconi@ars.toscana.it (P.F.); 2 Laboratorio Nazionale di Farmacologia e Medicina di Genere, Istituto Nazionale Biostrutture Biosistemi, University of Sassari, 07100 Sassari, Italy; franconi.flavia@gmail.com; 3 Diabetes and Metabolic Diseases Unit, Health Local Unit North-West Tuscany, 57121 Livorno, Italy; graziano.dicianni@uslnordovest.toscana.it

**Keywords:** gestational diabetes, risk factors, temporal trends, maternofetal adverse effects

## Abstract

**Background/Objectives:** This population-based observational study with a repeated cross-sectional design included singleton live births occurring in Tuscany, Italy, between 2010 and 2021, identified through regional Delivery Assistance Certificates (CeDAP) and linked with administrative healthcare databases. The study aimed to assess temporal trends in the prevalence of gestational diabetes mellitus (GDM) and to evaluate the contribution of major risk factors—particularly pregestational obesity, maternal age, parity, and origin from high-pressure migration countries (HPMC)—to GDM and related maternal–neonatal outcomes. The rationale for the study derives from the increasing worldwide prevalence of GDM, driven by changes in diagnostic criteria as well as evolving maternal demographic and metabolic risk profiles. **Methods:** The study included 266,394 singleton live births. GDM was identified using a previously validated algorithm based on insulin prescriptions, diabetes specialist visits, attendance to education programs, and postpartum oral glucose tolerance testing. Temporal trends in GDM, pregestational obesity, HPMC origin, and maternal–neonatal outcomes were assessed. Multivariable Poisson regression models with robust variance estimation were used to estimate adjusted prevalence ratios (PRs) and 95% confidence intervals (CIs) for GDM and adverse outcomes, including preterm birth, macrosomia, neonatal distress, and cesarean delivery. **Results:** During the study period, the annual number of births declined, whereas the overall mean prevalence of GDM was 11.37% and increased steadily from 8.23% in 2010 to 14.43% in 2021, corresponding to a relative increase of approximately 75%. Pregestational obesity and pregnancies among women from HPMC also increased over time. Multivariable analysis showed that pregestational obesity, advanced maternal age, multiparity, and HPMC origin were major independent determinants of GDM. Pregestational obesity emerged as the most important variable associated with both GDM and maternal–neonatal complications, including macrosomia, cesarean delivery, and neonatal distress, often showing stronger associations than GDM itself. Macrosomia and cesarean delivery declined over time, whereas preterm birth increased among pregnancies complicated by GDM. An inverse association between GDM and fetal macrosomia was observed in adjusted models, possibly reflecting effective clinical management, earlier delivery strategies, or algorithm-related selection effects. **Conclusions:** GDM prevalence increased substantially in Tuscany between 2010 and 2021 despite declining birth rates. This increase appears to be mainly associated with rising pregestational obesity, older maternal age, multiparity, and changes in population composition related to migration from HPMC. Adverse maternal–neonatal outcomes were more strongly associated with pregestational obesity and maternal age than with GDM itself, suggesting that preventive strategies targeting metabolic health before pregnancy may be crucial for reducing both GDM and related complications. Monitoring temporal trends in GDM and its risk factors may also serve as an indirect indicator of the effectiveness of regional healthcare policies.

## 1. Introduction

Epidemiological evidence indicates that the global prevalence of gestational diabetes mellitus (GDM) has steadily increased [1,2,3,4,5,6,7]. In any population, GDM risk is influenced by several well-established factors. In our setting, the most relevant are pregestational maternal obesity and maternal ethnicity, together with other factors such as advanced maternal age and parity [8,9,10]. In Tuscany, a region of central Italy where this study was conducted, the regional health service has long implemented guidelines and territorial care pathways for the management of GDM and its maternal–fetal complications, in accordance with Tuscany Regional Government Resolution No. 898/2012, “Percorso assistenziale e monitoraggio ostetrico del diabete gestazionale” [11]. This policy includes OGTT-based screening during pregnancy for the diagnosis of GDM and a network of outpatient gynecologists working closely with diabetologists to manage pregnancy from its early stages through delivery. This integrated setting provides an opportunity to monitor pre- and postpartum maternal–fetal outcomes in pregnancies complicated by GDM, which is associated with a wide range of adverse outcomes for both mothers and newborns [10,12,13,14]. A key issue in this context, and for the organization of healthcare policies for GDM, is the assessment of temporal changes in major risk factors, particularly pregestational obesity, as this may help clarify their contribution to parallel changes in GDM prevalence. Furthermore, in Tuscany, as in Italy overall, two major epidemiological trends have been observed in recent years: a progressive decline in the number of pregnancies and, at the same time, an increase in migrants from countries with a high prevalence of diabetes (High-Pressure Migration Countries; HPMC) [14,15,16]. Based on these considerations, and using regional administrative databases previously applied in Italy for similar purposes [17], this study focused on births in Tuscany from 2010 to 2021 and had three main aims: (i) to determine whether GDM prevalence increased during the study period; (ii) to assess whether this increase was accompanied by a rise in maternal–fetal complications; and (iii) to evaluate whether the association between GDM and adverse outcomes may be related to temporal changes in GDM risk factors.

## 2. Materials and Methods

This observational study with a repeated cross-sectional design included all pregnancies resulting in singleton live births between 1 January 2010 and 31 December 2021 among women aged 15–45 years, identified through the regional Delivery Assistance Certificates (Certificato di Assistenza al Parto, CeDAP). In Tuscany, CeDAP certificates are completed by midwives for nearly all pregnancies and record information on pregnancy, delivery, newborn characteristics, maternal age, pregestational body mass index (BMI), parity, education, and employment. Maternal citizenship was classified as Italian or non-Italian. The latter group was further categorized according to region of origin, including North Africa, Sub-Saharan Africa, South Asia, China, other Asian countries, Central or South America, and Eastern Europe. These regions were collectively referred to as high-pressure migration countries (HPMC) [15]. This acronym groups migrant women of different ethnic backgrounds from non-European countries, whose migration to Italy has increased substantially over the last decade. For the purpose of this study, women from some of these groups have, on average, a higher risk of GDM than Italian women [15].

### 2.1. Diagnosis of GDM

According to regional guidelines [11], all pregnant women are assessed for eligibility for screening with a 75 g-OGTT. Screening is recommended early in pregnancy (16–18 weeks) for women at high risk, including those with previous GDM, pregestational BMI ≥ 30 kg/m^2^, or fasting glucose at the first visit between 5.6 and 6.9 mmol/L. Screening is recommended later in pregnancy (24–28 weeks) for women at intermediate risk, including those aged > 35 years, those with pre-pregnancy BMI >25 and <30 kg/m^2^, previous macrosomia, a positive family history of type 2 diabetes mellitus, or ancestry from non-European countries with a high prevalence of diabetes. Low-risk women with no risk factors should be excluded from screening. The screening test was recommended for high- and intermediate-risk pregnancies, although previous evidence indicates that in Tuscany it was also administered to a substantial proportion of low-risk women [18]. From CeDAP data, GDM diagnosis followed a two-step OGTT strategy with a 75 g oral glucose load until late 2012 and IADPSG criteria thereafter, in accordance with national and regional guidelines [17,18,19]. For the present study, based on regional administrative datasets, women affected by GDM were identified using an algorithm [18] based on the fulfillment of at least one of the following criteria, considered strongly associated with GDM status: (a) women without prior antidiabetic therapy who were prescribed insulin—the only antidiabetic drug permitted during pregnancy in Italy according to guidelines—and discontinued it after delivery; (b) women who consulted a diabetes specialist or attended a diabetes education program before delivery; or (c) women who underwent an OGTT within six months after delivery. These data were obtained by linking the CeDAP source database, through a unique anonymized individual identification key, with other regional databases containing specialist visits, laboratory data, and drug-prescription claims [18].

The algorithm was validated in two independent cohorts, including a total of 5100 unselected pregnant women evaluated in 2014 and 2015 in one Local Health Authority of Florence, Italy. GDM status determined by the algorithm was matched, through the unique regional anonymized identification code, with GDM status established using standard OGTT glucose results. Accuracy measures were as follows: sensitivity, 0.460; specificity, 0.976; positive predictive value, 0.805 (95% CI: 0.774–0.836); and negative predictive value, 0.895. A similar validation procedure was conducted in a second cohort of 456 women from the Local Health Authority of Livorno, Italy, with the following accuracy measures: sensitivity, 0.520; specificity, 0.990; positive predictive value, 0.920; and negative predictive value, 0.910. Finally, an additional validation was performed by cross-referencing women identified as GDM-positive by the algorithm with those discharged from regional hospitals with an ICD-9 code associated with GDM (648.8), listed as either a primary or secondary diagnosis (No. = 17,606). Accuracy measures were as follows: sensitivity, 0.814; specificity, 0.911; positive predictive value, 0.252; and negative predictive value, 0.992 [18]. Women with pre-existing type 1 or type 2 diabetes were not included in this study.

### 2.2. Changes in Diagnostic Criteria over the Study Period

Following the Regional Government Resolution issued in October 2012 [11], the regional screening pathway was revised, moving from the previous two-step strategy to a one-step 75 g OGTT based on IADPSG criteria. Because the Tuscan public healthcare network accounts for approximately 90% of public maternal care and more than 95% of deliveries occur in public hospitals, 2013 should be considered a transition year, whereas the period from 2014 onward should be regarded as representative of full implementation of the IADPSG-based screening strategy.

### 2.3. Statistical Analysis

Univariate analyses were performed using standard methods. Time-trend analyses used the chi-square test for trend. For each year, the prevalence of major GDM risk factors—specifically pregestational obesity (BMI ≥ 30 kg/m^2^) and HPMC origin, both considered potential primary risk factors for GDM—was assessed in a comparable manner. Annual rates of maternal and neonatal outcomes, including preterm birth, neonatal macrosomia (birthweight ≥ 4000 g), neonatal distress defined as a 5-min-Apgar score ≤ 7, and cesarean delivery, were assessed across the entire study period.

Multivariable Poisson regression models with log link and robust variance estimation were used to estimate adjusted prevalence ratios (PRs) and 95% confidence intervals (95% CIs) for GDM, risk factors, and adverse maternal–neonatal outcomes (GENMOD procedure in SAS 9.3, Rev. 930_16w04). Calendar year was included as a continuous variable to assess temporal trends, whereas models were adjusted for maternal age, HPMC origin, parity, and pregestational obesity. Overdispersion was evaluated by inspecting the ratio of residual deviance to degrees of freedom. The deviance/df ratio ranged from 0.1969 to 0.7142 across models, indicating adequate fit and only mild overdispersion, corrected by the robust variance estimator. This approach was preferred over logistic regression because it allows direct estimation of PRs, which are easier to interpret in population-based studies with non-rare outcomes. Covariates included maternal age, modeled as a continuous variable; Italian or Western-country origin versus HPMC origin (1: Italy/Western countries, 0: HPMC countries); pregestational obesity; calendar year; and parity, coded as nulliparous (1) versus multiparous (0). Significant calendar-year effects in the models indicated the presence of either negative or positive temporal trends. Variables such as smoking, socioeconomic status, employment and education, although available in CeDAP certificates, were omitted from the analysis because of variable but consistent missingness, which markedly reduced the performance of all analyses. Missing data for variables included in the study were consistently below 10%.

This retrospective study was based on the linkage of regional healthcare pseudonymized databases. In accordance with Italian Legislative Decree No. 196/2003 (Personal Data Protection Code) and Regulation (EU) 2016/679 (General Data Protection Regulation, GDPR) on the protection of personal data, neither ethical committee approval nor informed consent was required. The study was conducted in compliance with the principles of the Declaration of Helsinki.

All analyses were conducted with SAS version. 9.3 and STATA 14. Statistical significance was set at *p* < 0.05.

## 3. Results

**Figure 1:**
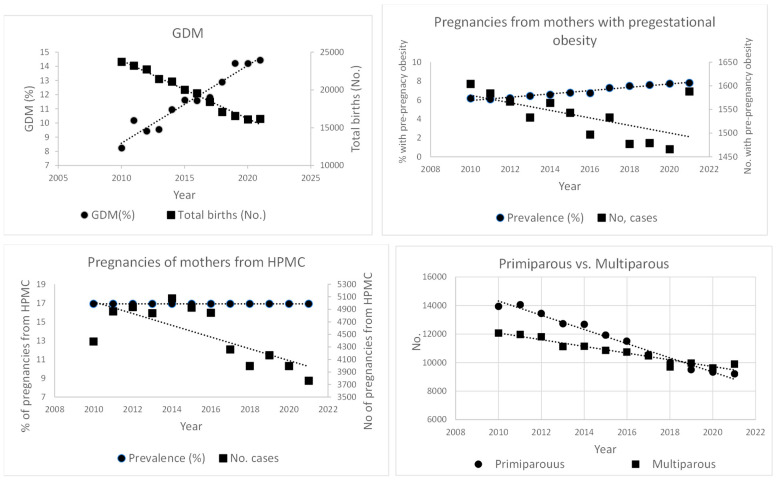
No. of cases and prevalence rates across years 2010–2021 in pregnancies in Tuscany with GDM, pregestational obesity, HPMC mothers and primiparous vs. multiparous. Dotted lines represent regression lines.

The study sample comprised 266,394 singleton pregnancies occurring in Tuscany, Italy, between 2010 and 2021. Table 1 summarizes the annual prevalence rates of the studied variables from 2010 to 2021. Over this period, the annual number of births progressively decreased, whereas the prevalence of GDM steadily increased (Table 1 and Figure 1). Similar upward trends were observed for pregestational obesity and for the annual prevalence of pregnancies among women from HPMC. Conversely, the number of primiparous women decreased consistently across the study years, while the number of multiparous women showed a similar, although more gradual, decline (Table 1 and Figure 1). Median maternal age and median neonatal birthweight remained stable over time (Table 1).

**Figure 2:**
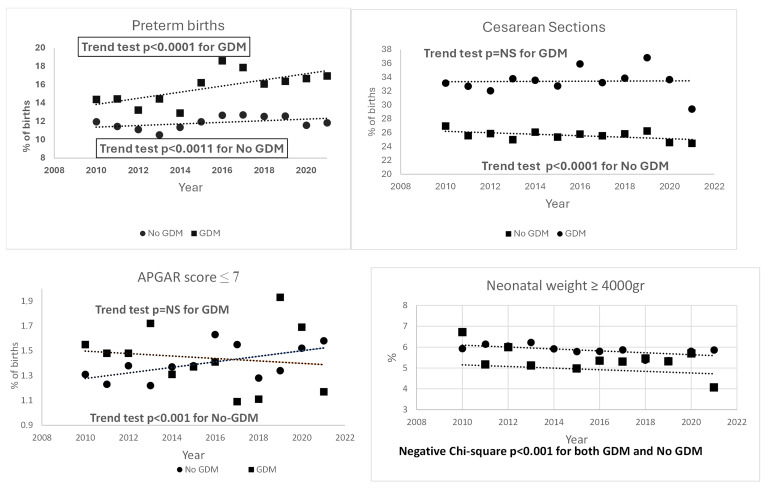
Annual trend of maternal fetal outcomes in Tuscany from 2010 to 2021. *Y*-axis percentages are calculated within each stratum (GDM vs. No GDM). Data were analyzed by Chi-square for trend.

The unadjusted univariate analysis of maternal and neonatal outcomes, stratified by GDM status, and evaluated by Chi-square trend test is shown in Figure 2.

In the GDM group, a marked yearly increase in the prevalence rate of preterm birth (≤37 weeks) was observed. In pregnancies without gestational diabetes, cesarean delivery decreased progressively, a trend that was not observed among pregnancies with GDM. The annual prevalence of macrosomia decreased in pregnancies with and without GDM. At the same time, there was a slight increase in newborns with Apgar scores ≤ 7 among pregnancies without GDM, whereas this trend was not observed in the GDM cohort.

Table 2 summarizes the multivariable Poisson regression results. GDM and pregestational obesity increased independently over time, with annual PRs indicating an approximately 4% rise during the study period. Conversely, fetal macrosomia (≥4000 g) and cesarean delivery decreased by about 1–2% per year. The prevalence of Apgar score ≤ 7 remained largely unchanged, showing only a small annual increase (PR: 1.015; 95% CI: 1.005–1.024; *p* = 0.0001), and was not significantly associated with GDM. Maternal age showed modest but significant associations with GDM, cesarean delivery, neonatal distress, and preterm birth. Pregestational obesity was the strongest determinant of GDM, nearly doubling its prevalence, and was also strongly associated with all adverse outcomes considered. Its associations with cesarean delivery, neonatal macrosomia, and neonatal distress were stronger than those observed for GDM. Multiparity was associated with pregestational obesity, GDM, and fetal macrosomia. Fetal macrosomia was mainly driven by multiparity and pregestational obesity, whereas GDM was inversely associated with this outcome (PR = 0.854; 95% CI: 0.810–0.900; *p* < 0.0001). Compared with women from HPMC, women of Italian origin had significantly lower PRs for all outcomes examined. GDM independently predicted preterm birth, whereas Apgar score ≤ 7 was mainly associated with maternal age, pregestational obesity, primiparity, and HPMC origin, but not with GDM.

## 4. Discussion

Recent European estimates indicate that GDM affects approximately 10–14% of pregnancies, although prevalence varies substantially according to diagnostic criteria, screening strategies, maternal age, obesity, the prevalence of migrant mothers at higher risk of GDM, and population composition. In a European meta-analysis, the overall prevalence was 10.9%, with higher estimates in Southern Europe [20]. In Italy and Southern Europe, available regional studies report heterogeneous values, ranging from approximately 6% to 12% [5,6,18]. Further marked differences among reported estimates are largely attributable to different data sources, including administrative databases, hospital-based clinical series, and hospital discharge records coded with ICD-9 codes. However, supporting the validity of the algorithm used in our study, a systematic review of Italian healthcare administrative databases for epidemiological studies of diabetes confirms that our algorithm is substantially aligned with those used in other epidemiological investigations [17]. It should be noted, however, that the moderate sensitivity of the algorithm (0.46–0.52 in the two cohort validations) implies that the prevalence estimates reported here likely underestimate true GDM occurrence. In conclusion, the mean GDM prevalence observed in our study between 2010 and 2021 (11.37%) is broadly consistent with European and Southern European data [20], while the increase from 8.23% to 14.43%, corresponding to a relative increase of approximately 75% over the study period, confirms an upward temporal trend. As detailed in the Methods section, in Tuscany, GDM screening is performed using a 75 g-OGTT according to IADPSG criteria, in accordance with Italian national guidelines implemented in our region in late 2012, either between 24 and 28 weeks of gestation or earlier (16–18 weeks) in the presence of major risk factors [18]. Our analysis of temporal trends from 2010 to 2021 confirms a progressive increase in GDM cases over time. Possible drivers of this increase include adoption of the more sensitive IADPSG criteria, although in our setting these were applied only from late 2012 onward. The transient decline in GDM prevalence in 2012, followed by a sustained rise from 2013 onward, is consistent with the progressive implementation of IADPSG criteria described in Section 2.2, with 2013 representing a transition year and full adoption consolidated from 2014. Additional contributing factors may include the increasing prevalence of maternal pregestational obesity and the growing number of pregnancies among women from HPMC. In the latter case, the increase may be partly attributable to pregnancies among migrant women from regions at higher risk of GDM, such as North Africa, Southeast Asia, and Eastern Europe [15,21]. Nevertheless, it is difficult to quantify the relative contribution of each of these factors to the increase in GDM incidence over time, although it should be considered that this is a global phenomenon observed worldwide in recent years [1,2,3,4,5,6,7]. The key finding of our study, consistent with previous evidence, is that the temporal increase in GDM prevalence can largely be attributed to the concurrent rise in pregestational obesity, older maternal age, multiparity, and the increasing number of births among women from HPMC. This conclusion is supported by both univariate analyses and multivariable Poisson regression models. The mean age at motherhood has been increasing in Western countries. In Italy, national birth registry data from the Ministry of Health based on CeDAP certificates show that the mean age of first-time mothers increased from 31.5 years in 2012 to over 32 years in 2022 [22]. This reflects a consistent trend toward later childbearing in Italy, which is also applicable to Tuscany. Furthermore, as a proof of concept, we found that women with GDM tended to be older than those without GDM.

We also observed a progressive decline in births among primiparous women, a trend that may influence analyses of parity-related GDM risk. Evidence regarding parity as a risk factor for GDM is mixed; however, most studies suggest that multiparity is associated with an increased risk of GDM, likely because of the progressive increase in insulin resistance across repeated pregnancies [23,24,25]. In the present study, pregestational obesity was a significant independent multiplier of GDM prevalence over time. This interpretation is consistent with the meta-analysis by Chu et al., published in Diabetes Care, showing a graded association between increasing pre-pregnancy BMI and the risk of GDM, thereby supporting pregestational obesity as one of the strongest modifiable determinants of GDM [9], as well as of associated complications, including macrosomia, neonatal distress, and cesarean delivery [26,27,28]. Interestingly, the temporal trend in obesity prevalence was significantly associated with Italian origin compared with HPMC origin (PR = 1.04; 95% CI: 1.004–1.083; *p* = 0.03), indicating that women from HPMC were not, on average, more obese than Italian women. Although, by its design this study lacks data on metabolic control or maternal blood glucose levels in GDM, these findings suggest that pregestational obesity may have a stronger impact on neonatal macrosomia than maternal hyperglycemia itself, at least within this healthcare setting. As previously reported, maternal blood glucose fluctuations account for approximately 2–13% of birthweight variance, particularly when pregnancy is uncomplicated or GDM is effectively controlled [29,30,31]. Maternal obesity may also exert adverse effects on offspring, as suggested by a recent Chinese study showing that maternal overweight and GDM, separately and jointly, are associated with rapid increases in adiposity from birth through early adolescence [28]. Finally, multivariable analysis showed that Italian origin, compared with HPMC origin, was associated with lower PRs for GDM, macrosomia, and preterm birth. This may be partly explained by disparities in access to healthcare services and differences in the implementation of regional policies for GDM screening and management among migrant populations, including HPMC migrants within our region [18], seemingly independent of pregestational obesity which is more strongly associated with Italian ancestry. Cesarean delivery and macrosomia decreased over time in non-GDM pregnancies and, according to adjusted multivariable models, also among pregnancies with GDM. These findings may indirectly suggest that regional health policies have contributed to improved early detection and prevention of adverse outcomes associated with GDM, as well as to more judicious use of cesarean delivery. Preterm births increased among GDM pregnancies. This increase may partly reflect an intentional clinical strategy to anticipate delivery in order to reduce the risk of macrosomia and more severe late-pregnancy glycemic imbalance, although data on obstetric indications for preterm delivery were not available in this dataset. Univariate analysis showed a reduction in neonatal distress (Apgar score ≤ 7) over time within the GDM group, although the total number of affected newborns remained persistently low. These findings further suggest effective management by healthcare professionals and facilities in our region.

An intriguing finding of this study is the inverse association between GDM and the prevalence of fetal macrosomia. In this regard, a recent study conducted in Southern China reported a decline over time in the prevalence of macrosomia among pregnancies with GDM, probably reflecting improvements in metabolic control in women with GDM over the years [32]. Moreover, although rates of fetal overweight remained somewhat elevated in that population, this finding again suggests distinct contributions of glucose levels and other factors, including maternal obesity, to neonatal weight. In addition, elective early delivery in pregnancies complicated by GDM may contribute to the reduced occurrence of macrosomia. According to a second hypothesis, the algorithm may preferentially capture more clinically complex cases, suggesting the possibility that the healthcare system pays particular attention to macrosomia in pregnancies with GDM. The alternative hypothesis of a common mediating role of pregestational obesity in both GDM and macrosomia, resulting in collinearity, appears unlikely because of their divergent associations with the PR for macrosomia, further reinforcing the counterintuitive nature of the inverse association between GDM and fetal macrosomia. Finally, findings from the multivariable analysis again show that maternal age plays an important and often underestimated role in GDM, pregestational obesity, and neonatal complications.

### Limitations and Strengths of the Study

This study has several important limitations. First, it is based on administrative databases, and the diagnosis of GDM relies on an algorithmic approach which, although previously validated and applied in a region with universal GDM screening, does not include relevant information on metabolic control during pregnancy. The change in the GDM screening strategy at the end of 2012, from a two-step 75 g OGTT-based approach to the one-step IADPSG method, may have increased GDM rates over time [33]. This represents an additional limitation in suggesting a relative increase in GDM prevalence; however, it is unlikely to invalidate the overall increasing temporal trend in GDM prevalence across the entire observation period. A further limitation is that CeDAP certificates do not contain data on stillbirths or late pregnancy losses, which may underestimate the burden of adverse outcomes associated with obesity or GDM. The lack of reliable data on employment status or education, owing to substantial and non-homogeneous missingness over time, represents another limitation. An additional limitation is related to the repeated cross-sectional observational design of the study, which limits causal inference and the generalizability of the findings to other populations. Finally, the moderate sensitivity of the algorithm (0.46–0.52 in the two cohort validations reported in Methods) implies that the prevalence estimates reported here likely underestimate true GDM occurrence. Reported associations should therefore be interpreted as reflecting algorithmically identified GDM, which may be enriched for clinically overt cases. If non-capture rates differ between Italian and HPMC women—for instance, because of differential use of diabetes specialist services—the HPMC coefficient may be affected by differential misclassification.

The strengths of this study include the large sample size, the long enough observation period, the methodological consistency maintained throughout the study, and the near complete coverage of all pregnancies occurring in Tuscany between 2010 and 2021.

## 5. Conclusions

First, the prevalence of GDM, as captured here through administrative databases, is consistent with expected prevalence estimates from other studies conducted in Southern Europe, and, even considering alla limitations, a true temporal increase in GDM prevalence also appears to have occurred in our region, in line with trends observed worldwide. This increase occurred despite a progressive decline in regional birth rates. Second, this study confirms that pregestational obesity, maternal origin from HPMC, advanced maternal age, and multiparity are the main drivers of the temporal increase in GDM prevalence. Third, the study shows that maternal–fetal complications, such as fetal macrosomia, cesarean delivery, and neonatal distress, are primarily associated with pregestational obesity and maternal age rather than with GDM itself. These findings therefore support the need for obesity prevention policies aimed at reducing both GDM and its adverse outcomes in our population. They also highlight that prevention of obesity in youth may reduce the risk of GDM, interrupt the intergenerational transmission of metabolic risk, and help preserve long-term cardiometabolic health [34]. A further often underestimated policy objective should be the implementation of measures that support pregnancy at younger ages. The intriguing inverse association between GDM and lower PRs of fetal macrosomia may be related to policies favoring earlier delivery in these cases. According to a second hypothesis, the algorithm may preferentially capture more clinically complex cases, suggesting that the healthcare system pays particular attention to macrosomia in pregnancies with GDM. This finding may indicate that Tuscany has achieved good standards in the management of GDM and its adverse outcomes. In summary, monitoring temporal trends in GDM and its related risk factors may serve as an indirect indicator of the effectiveness of regional healthcare policies. This process may help identify policies that improve prevention of GDM and its adverse maternal and neonatal outcomes, while also highlighting where broader prevention interventions are most needed, particularly against youth obesity.

## Figures and Tables

**Table 1:** Characteristics of the population under study. Rates in parentheses represent No. of cases/total pregnancies.

Year	2010	2011	2012	2013	2014	2015	2016	2017	2018	2019	2020	2021	Total
Pregnancies (No.)	25,842	25,841	25,089	23,705	23,697	22,652	22,122	20,894	19,604	19,294	18,765	18,889	266,394
GDM (No.%)	2127 (8.23)	2630 (10.18)	2365 (9.43)	2264 (9.55)	2594 (10.95)	2632 (11.62)	2559 (11.57)	2468 (11.81)	2527 (12.89)	2742 (14.21)	2665 (14.20)	2726 (14.43)	30,299 (11.37)
Median maternal age (yr)	32	32	32	32	32	32	32	32	32	32	32	33	32
Primiparous (No.%)	13,942 (53.60)	14,062 (54.02)	13,447 (53.23)	12,730 (53.36)	12,688 (53.24)	11,991 (52.35)	11,511 (51.72)	10,560 (50.21)	10,020 (50.81)	9512 (48.83)	9339 (49.24)	9216 (48.22)	138,958 (51.78)
Median neonatal weight (g)	3290	3280	3290	3290	3290	3280	3280	3290	3280	3280	3290	3290	3290
Migrants From HPMC (No., %)	4386 (16.92)	4868 (18.78)	4934 (19.59)	4838 (20.31)	5074 (21.30)	4926 (21.62)	4844 (21.79)	4260 (20.28)	3996 (20.28)	4168 (21.40)	3995 (21.07)	3761 (19.77)	54,050 (20.29)
Pregestational BMI ≥ 30 kg/m^2^ (No.%)	1604 (6.17)	1584 (6.08)	1567 (6.20)	1533 (6.43)	1564 (6.56)	1543 (6.77)	1497 (6.73)	1533 (7.29)	1477 (7.49)	1479 (7.59)	1466 (7.73)	1588 (8.31)	18,435 (6.87)
Cesarean Section (No.%)	7193 (27.8)	6890 (26.47)	6734 (26.67)	6204 (26.00)	6441 (27.03)	6016 (26.40)	6047 (27.17)	5592 (26.59)	5337 (27.07)	5453 (27.99)	4955 (26.13)	4857 (25.41)	71,719 (26.73)
Preterm birth (No., %)	3202 (12.31)	3095 (11.89)	2898 (11.47)	2627 (11.21)	2771 (11.63)	2868 (12.58)	3004 (13.50)	2833 (13.47)	2599 (13.18)	2591 (13.30)	2365 (12.47)	2451 (12.82)	33,304 (12.41)
Neonatal weight ≥ 4000 g (No. %)	1561 (6.00)	1577 (6.06)	1526 (6.04)	1458 (6.11)	1405 (5.90)	1310 (5.75)	1280 (5.75)	1224 (5.82)	1063 (5.39)	1043 (5.35)	1101 (5.81)	1055 (5.57)	15,613 (5.82)
Apgar score ≤ 7 (No.%)	350 (1.35)	328 (1.26)	353 (1.40)	308 (1.29)	328 (1.38)	316 (1.39)	359 (1.61)	317 (1.51)	253 (1.28)	280 (1.44)	293 (1.54)	295 (1.54)	3780 (1.41)

**Table 2:** Multivariate analysis by Poisson models with Prevalence Ratios (PR) and 95% CI for each independent variable entering the model and with the same panel of covariates (* Italian/western countries = 1, HPMC countries = 0; ** Primiparous = 1, multiparous = 0).

**GDM**			
	**PR**	**95% CI**	***p***
Age	1.062	1.059–1.064	<0.0001
Italian vs. HPMC *	0.651	0.633–0.669	<0.0001
Calendar year	1.043	1.040–1.047	<0.0001
Primiparous **	0.923	0.902–0.946	<0.0001
Pregest. obesity	2.337	2.260–2.410	<0.0001
**Cesarean Sections**			
	**PR**	**95% CI**	***p***
Age	1.051	1.049–1.052	<0.0001
Italian vs. HPMC *	0.882	0.865–0.900	<0.0001
Calendar year	0.995	0.993–0.997	<0.0001
Primiparous **	1.151	1.133–1.169	<0.0001
Pregest. obesity	1.453	1.399–1.472	<0.0001
GDM	1.163	1.138–1.188	<0.0001
**Preterm Delivery (≤37 weeks)**			
	**PR**	**95% CI**	***p***
Age	1.032	1.030–1.035	<0.0001
Italian vs. HPMC *	0.793	0.770–0.815	<0.0001
Calendar year	1.008	1.005–1.011	<0.0001
Primiparous **	1.115	1.009–1.141	<0.0001
Pregest. obesity	1.101	1.056–1.148	<0.0001
GDM	1.243	1.205–1.283	<0.0001
**Pregestational Obesity**			
	**PR**	**95% CI**	***p***
Age	0.995	0.992–0.996	0.0009
Italian vs. HPMC *	1.043	1.004–1.083	0.0300
Calendar year	1.026	1.022–1.031	<0.0034
Primiparous **	0.723	0.701–0.745	<0.0001
**Neonatal Weight ≥ 4000 g**			
	**PR**	**95% CI**	***p***
Age	0.994	0.991–0.997	0.0008
Italian vs. HPMC *	0.839	0.807–0.873	<0.0008
Calendar year	0.987	0.983–0.992	<0.0001
Primiparous **	0.643	0.621–0.665	<0.0001
Pregest. obesity	1.700	1.616–1.789	<0.0001
GDM	0.854	0.810–0.900	<0.0001
**Apgar Score ≤ 7**			
	**PR**	**95% CI**	***p***
Age	1.025	1.018–1.032	<0.0001
Italian vs. HPMC *	0.734	0.676–0.797	<0.0001
Calendar year	1.015	1.005–1.024	<0.0001
Primiparous **	1.556	1.452–1.667	<0.0001
Pregest. obesity	1.498	1.339–1.675	<0.0001
GDM	0.946	0.854–1.049	NS

## Data Availability

The original contributions presented in this study are included in the article. Further inquiries can be directed to the corresponding author.
